# Delayed Presentation of Popliteal Artery Injury after Salter-Harris III Proximal Tibia Fracture

**DOI:** 10.1155/2023/4104127

**Published:** 2023-12-05

**Authors:** Alexandra H. Seidenstein, Timothy W. Torrez, Jacob A. Garcia, Shadi K. Awad, Henry Debell, Shawn R. Gilbert, Kevin A. Williams

**Affiliations:** ^1^Department of Orthopaedic Surgery, University of Alabama at Birmingham Heersink School of Medicine, Birmingham, AL 35233, USA; ^2^Department of Orthopaedic Surgery, University of Alabama at Birmingham Heersink School of Medicine, Children's of Alabama, Birmingham 35233, USA

## Abstract

**Introduction:**

Proximal tibia physeal fractures in children are not very common but can be dangerous because they can harm popliteal fossa structures, especially the popliteal artery. Popliteal artery injuries (PAI) are most commonly the result of trauma to the lower extremity, including blunt force, hyperextension injuries, complex fractures, and knee dislocations that can compromise popliteal neurovascular structures. *Case Presentation.* A 14-year-old boy presents to the emergency department after being transferred from an outside hospital 24 hours after a left lower extremity hyperextension injury. Radiographs demonstrated a Salter-Harris III proximal tibia fracture with posterior displacement. ABIs were deferred due to palpable distal pulses and no evidence of compartment syndrome. Closed reduction and percutaneous pinning were planned to correct the fracture. Intraoperatively, it was discovered that knee extension decreased lower extremity perfusion while knee flexion returned perfusion. An angiography revealed a popliteal artery occlusion with no distal flow. Based on this, an above-knee to below-knee popliteal bypass using the contralateral great saphenous vein was performed followed by closed reduction and percutaneous pinning of the proximal tibia.

**Conclusion:**

Proximal tibia physeal injuries, especially the Salter-Harris III and IV injuries, warrant a high index of suspicion of popliteal artery injuries. Palpable pulses and delayed presentation in the distal lower extremity do not rule out a PAI because collateral flow to the anterior and posterior tibial arteries may mask signs of an avascular limb, highlighting the need for a thorough history and physical exam. The authors present this case to reaffirm the importance of an ankle-brachial index when evaluating hyperextension injuries with proximal tibial epiphyseal fractures.

## 1. Introduction

Despite tibial fractures accounting for 15% of all pediatric fractures, proximal tibia physeal injuries occur in only 0.5-3% of all long bone fractures [1, 2]. Popliteal artery injuries can accompany adolescent lower extremity fractures. Maithel et al. noted significantly increased odds of developing PAI following fractures of tibia, fibula, femur, and patella in comparison to other lower extremity trauma [3]. Vascular injuries in the setting of pediatric trauma are relatively rare, accounting for between 0.6 and 1.4% of injuries [4]. Of those instances of trauma-related vascular injuries, only 5% of those are PAI [2, 5]. PAI is most commonly secondary to blunt and penetrating trauma to the lower extremity [3].

The anatomical location of the popliteal fossa, nestled deep behind the knee joint, inherently offers some protection to its neurovascular structures. However, this protection is not absolute; blunt trauma, hyperextension injuries, complex fractures, and knee dislocations can still compromise and injure these critical elements [2, 6, 7]. In this case report, we present a patient with a Salter-Harris III (SHIII) proximal tibia fracture and a delayed presentation of an associated popliteal artery injury that likely occurred secondary to a hyperextension injury during a wrestling match.

There are few limited case reports involving PAI with concurrent physeal fractures. Previous literature only describes type-1 SH fractures resulting in PAIs. The patient was an 11-year-old female, with a SHI distal femur fracture caused by her right leg being caught in a swing. Treatment consisted of same-day closed reduction and fixation. About 24 h later, there was hypoesthesia followed by decreased toe movement and loss of pulses. Angiogram revealed a lower extremity thrombosis, and fasciotomies were performed. Four-year outcomes demonstrated continued dysfunction of the patient's limb despite the treatment [5].

These case reports highlight the difficulty in diagnosing PAI, as well as their associated immediate and long-term outcomes. Quick vascular assessment is paramount, and clinicians should have increased suspicion of PAI. Although rare, this should be considered in all pediatric cases involving trauma around the knee [5]. Our case report of PAI in conjunction with SH-III injury is unique in that the patient did not present until 24 h had passed from the initial trauma, and peripheral pulses were present upon initial examination. In addition, the patient's tenuous perfusion due to collateral blood flow was positional, and the patient experienced loss of blood flow with knee extension. The delay in presentation of the PAI and subtle signs of ischemia reinstate the need for clinicians to be aware and fully evaluate for PAI in knee injuries with additional workup.

## 2. Case Presentation

**Figure 1:**
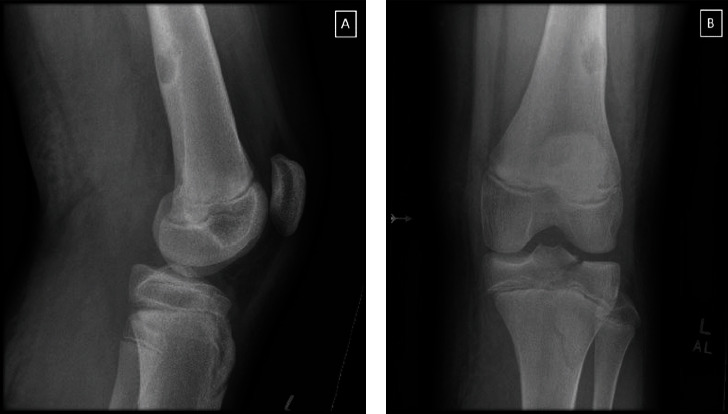
Knee radiograph demonstrating a displaced Salter-Harris III fracture of the proximal tibial epiphysis with incidental benign cortical lesion in the distal femur. (a) Lateral; (b) anterior posterior.

The patient is a 14-year-old male with a delayed presentation of a popliteal artery injury associated with a hyperextension injury. The patient felt an immediate pop in his knee during a wrestling match with subsequent pain and inability to bear weight. He denied any deformity or dislocation at the time of the injury. At home, he noted significant swelling and positional paresthesia, but he and his parents were initially reluctant to present for further evaluation. He presented to an outside hospital the following afternoon due to continued pain, inability to bear weight, worsening swelling, and posterior thigh ecchymosis after elevating and icing his injured extremity overnight. He was transferred to our institution roughly 24 h after the injury with concerns for compartment syndrome, due to increased pain to mild motion, and a proximal tibia fracture. Upon admission, radiographs demonstrated a SHIII proximal tibia fracture with approximately 5 mm of posterolateral displacement of the medial fragment (Figure 1).

Physical exam demonstrated popliteal ecchymosis, palpable posterior tibial and dorsalis pedis pulses, brisk capillary refill, weakness in dorsiflexion that was thought to be secondary to pain, and no evidence of compartment syndrome. Evaluation of ankle-brachial indices (ABIs) was deferred given the patient's delayed presentation of roughly 24 h, palpable pulses, and apparent perfusion distal to the injury. An arthroscopically assisted closed reduction and percutaneous pinning of the fracture was planned for the following morning. He was serially examined overnight without change. During surgical preparation, the patient's operative limb was extended without hyperextension or dislocation. Extension of the knee resulted in an apparent change in perfusion with discoloration, decreased temperature, and loss of palpable pulses. The patient's leg was placed back into the prior resting position with thirty degrees of knee flexion, and the temperature and color returned to the extremity. Following this rapid sequence of events, the patient's lower extremity was examined with a Doppler, but no signal was found. Ultrasonography confirmed that there was blood flow distal to the popliteal fossa, but there was decreased flow compared to the contralateral side. The decision was then made to immediately consult vascular surgery. Due to lack of vascular surgery equipment in the pediatric operating rooms, the patient was transferred through the hallway to the adult hospital under anesthesia for angiography at the recommendation of vascular surgery. After roughly thirty minutes, a dorsalis pedis pulse was found on the Doppler, and the foot remained warm with brisk capillary refill. Contralateral pulses were palpable at this time.

**Figure 2:**
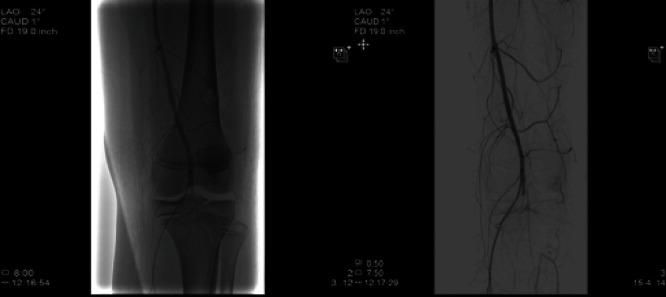
Arteriogram demonstrating occlusion of the popliteal artery at the level of the SHIII fracture of the proximal tibial epiphysis.

**Figure 3:**
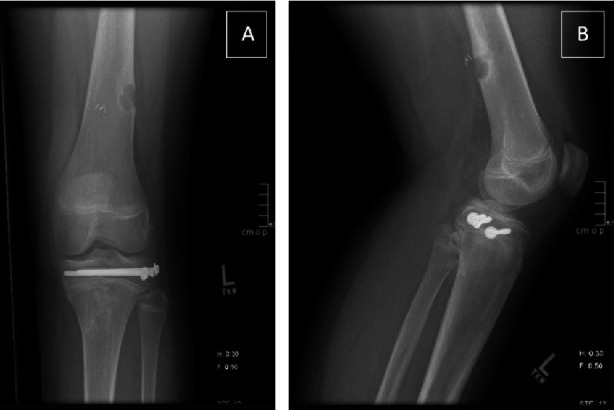
Postoperative knee radiograph demonstrating screw fixation with anatomic reduction and healing of displaced proximal tibial epiphyseal with metallic clips from vascular surgery. (a) Lateral; (b) anterior posterior.

**Figure 4:**
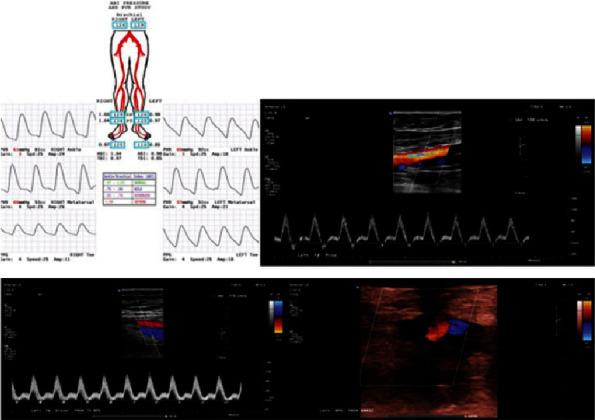
Color Doppler ultrasound/ABI obtained 2 months status postoperative intervention indicating graft patency in both longitudinal and transverse planes.

Vascular surgery performed angiography roughly four hours after the transient ischemic episode. This demonstrated a patent left common femoral artery, profunda, and superficial femoral artery above the knee. There was an occlusion at the level of the popliteal artery with no distal flow through the artery. There was reconstitution of the anterior tibial and posterior tibial arteries due to apparent collateral flow (Figure 2). Flow was present but limited to the foot. This collateral flow likely contributed to his delayed presentation and lack of concerning signs of an avascular limb. Based on these findings, the decision was made to perform a left above-knee to below-knee popliteal artery bypass using contralateral great saphenous vein. He also underwent prophylactic four-compartment fasciotomies. After completion of the vascular procedures, all incisions were closed except for the fasciotomy sites. The patient then underwent closed reduction and percutaneous pinning of the proximal tibia. Fluoroscopic guidance was used to place three 4.5 mm cannulated screws from lateral to medial through the epiphysis while maintaining reduction with a clamp (Figure 3). He was then placed in a knee immobilizer to protect his articular reduction and vascular bypass. The injured portion of the vessel was never explored or directly visualized, and the exact vascular injury mechanism remains unclear at this time. A six-week follow-up appointment with the color Doppler showed continued patency of the SVG graft (Figure 4).

In total, the patient received the following surgical procedures due to his SHIII fracture: left lower extremity angiogram, left four compartment prophylactic fasciotomies, right saphenous vein graft harvesting, left lower extremity above-knee to below-knee popliteal artery bypass, closed reduction and percutaneous pinning of the left proximal tibia SHIII fracture, and irrigation and debridement with closure of left foreleg fasciotomy incisions on postoperative day three.

The patient was admitted to the intensive care unit following an uneventful postoperative course and was transferred back to the pediatric hospital on postoperative day four. He was discharged on postoperative day six. Of note, his discharge was likely delayed due to a positive COVID-19 test on postoperative day five. The patient was prescribed an anticoagulation regiment of enoxaparin 40 mg twice daily and indefinite aspirin 81 mg daily. All graft management was handled by the vascular team. The patient's presentation of foot drop and paresthesia in the peroneal nerve distributions began to improve two weeks postoperatively and completely resolved after four weeks.

## 3. Discussion

This case provides a clear demonstration of a popliteal artery injury associated with a SHIII proximal tibia fracture. The assumed mechanism of injury was hyperextension and rotation on a fixed lower leg, with additional body mass of another competitor during a wrestling match. The supporting collateral and capsular ligaments are generally sufficient to maintain adequate support to the peripheral epiphysis and shield it from damage because of shear or translational force [2]. Hyperextension injuries are the most common cause of SHIII fractures of the proximal tibia, leading to posterior displacement of the tibial shaft relative to the epiphysis [2]. This type of trauma can be complicated by ligamentous injuries, vascular injuries, compartment syndrome, knee instability, and growth disturbances.

Previous research has shown that popliteal artery injuries are extremely rare; in fact, Meagher et al. reported on 53 pediatric vascular injuries resulting from blunt and penetrating trauma, none of which involved the popliteal artery, further confirming the low incidence of this injury. These articles reinforce the rarity of PAI injuries in the pediatric population, while highlighting the potential detrimental outcomes and the need for rapid detection. Jones et al. report three PAIs, one of which was caused by a physeal injury. Yadav et al. describe a displaced SH-I proximal tibia fracture with compromised vascularity of the lower limb. Immediate surgical repair to the fracture was carried out, which led to complete restoration of the vascularity of the limb. The patient went on to have functional recovery at 1-year follow-up [8]. Lastly, Shinomiya et al. reported a rare case of delayed PAI in a 14-year-old female after a hyperextension injury sustained from jumping off a horse. The patient was initially misdiagnosed with a gastroc-soleus injury when a proximal tibial physeal fracture was missed on standard radiographs. Additionally, both the dorsalis pedis and the posterior tibial arteries were palpable on arrival. The PAI and SH1 fractures were subsequently diagnosed by CT and Doppler US after the progression of pain, swelling, and decreased sensation to the calf and foot led to hospital transfer about 11 h later. At 1-year follow-up, this patient's outcome was a bony union, full range of motion, and a mild sensory deficit in the dorsum of the right foot [9].

In our case, it is likely that there was a significant amount of tension placed on the soft tissues of the posterior knee and the popliteal neurovascular structures, accentuating the tether associated with the artery's anatomic location adjacent to the proximal tibial physis and causing the PAI. The increased symptom severity prior to the patient's presentation likely indicated popliteal artery insufficiency. The collateral flow distal to the vascular injury likely contributed to his subacute presentation as he did not have a completely avascular limb. Regardless of the timing of presentation, the course of this patient's injury highlights the importance of achieving an adequate ABI to assess for any potential perfusion deficiencies in any pediatric trauma around the knee. This also demonstrates that palpable or dopplerable pulses may not be sufficient to rule out a potential arterial injury [10]. A delay in diagnosing a popliteal artery injury can have many ramifications including but not limited to limb ischemia, tissue necrosis, and delayed treatment. Rapid identification and urgent vascular consultation are paramount for the prevention of prolonged ischemia and limb preservation. Given the stakes, first responders, athletic trainers, and clinicians should be vigilant in advocating for timely and thorough evaluations, including ABI measurements, in pediatric patients presenting with knee trauma.

Unlike popliteal injuries seen in adults, pediatric PAI can commonly present with delayed onset of symptoms. This can result in increased time to diagnosis which puts lower extremity tissue at ischemic risk.^3^ Collateral blood flow from the popliteal artery is often limited which causes a PAI to be a very severe limb-threatening injury [3]. PAI is associated with an alarming rate of above- and below-the-knee amputations, accounting for 3-10% of cases [3, 11]. This increase in severity is due to the pediatrics population increased likelihood of sustaining a physis injury, rather than a simple ligamentous injury after trauma. Additionally, PAI is associated with a high rate of distal perfusion deficits, resulting in ischemic damage to the distal soft tissues. These injuries are difficult to manage, and limited data is available due to their rare incidence [12]. The most common methods of treatment in pediatric patients suffering PAI were *via* open procedures (62.1%), followed by nonoperative (36.2%) and endovascular-type procedures (10%) [3]. Our patient received an open bypass procedure.

High-grade physeal injuries, particularly types III and IV, are more commonly associated with higher morbidity secondary to increased complication rates such as physeal disturbances, ligamentous instability, and compartment syndrome. Proximal tibia SH III and IV injuries are more commonly seen in older adolescents with hyperextension injuries such as in our patient [13–16]. Involvement of the articular cartilage can result in additional pathology such as advancing osteoarthritis [13]. These increased complications can necessitate future surgical intervention. At the most recent follow-up, this patient exhibits no signs of physeal arrest or growth disturbance.

## Data Availability

The data used to support the findings in this study are included within the article.
